# Relationship Between Heart Disease and Obesity Indicators Among Adults: A Secondary Data Analysis

**DOI:** 10.7759/cureus.36738

**Published:** 2023-03-27

**Authors:** Khalid S Alwadeai, Mohammed A Almeshari, Abdulrahman S Alghamdi, Abdulrahman M Alshehri, Sulaiman S Alsaif, Muhammad O Al-Heizan, Mesfer S Alwadei, Ayedh D Alahmari, Saleh S Algarni, Tareq F Alotaibi, Mohammed M Alqahtani, Naji Alqahtani, Jaber S Alqahtani, Abdulelah M Aldhahir, Mazen M Homoud, Saad A Alhammad

**Affiliations:** 1 Department of Rehabilitation Sciences, College of Applied Medical Sciences, King Saud University, Riyadh, SAU; 2 Department of Radiology, Dhurma General Hospital, Dhurma, SAU; 3 Department of Respiratory Therapy, Batterjee Medical College, Jeddah, SAU; 4 Department of Respiratory Therapy, King Saud bin Abdulaziz University for Health Sciences, Riyadh, SAU; 5 Department of Research, King Abdullah International Medical Research Center, Riyadh, SAU; 6 Department of Respiratory Services, King Abdulaziz Medical City, Riyadh, SAU; 7 Department of Nursing Administration and Education, King Saud University, Riyadh, SAU; 8 Department of Respiratory Care, Prince Sultan Military College of Health Sciences, Dammam, SAU; 9 Department of Respiratory Therapy, Jazan University, Jazan, SAU; 10 Department of Respiratory Therapy, King Abdulaziz University, Riyadh, SAU

**Keywords:** lifestyle, sociodemographic, waist, obesity, heart disease

## Abstract

Background

Body mass index (BMI), waist circumference (WC), and hip circumference (HC) determine obesity. Several studies have examined the association between obesity and many diseases, including heart disease, and found it to be a substantial risk factor. However, the relationship between heart disease and obesity has not been investigated. This study investigated the relationship between heart disease and obesity indicators among adults encompassing sociodemographic and lifestyle factors.

Methodology

This cross-sectional study included data from 3,574 individuals who participated in the 2011-2014 National Survey of Midlife Development in the United States refresher. The presence or absence of heart conditions such as irregular heartbeat, heart murmur, heart attack, and heart failure was determined using self-reported questionnaires. The association between heart disease and obesity indicators such as BMI, WC, HC, and waist-to-hip ratio (WHR) was investigated using linear regression.

Results

After controlling for all factors, the findings demonstrated a significant relationship between heart disease and BMI, WC, and HC high scores of 1.12 kg/m^2^, 0.63 inches, and 0.81 inches, respectively. A higher score in all obesity indicators was linked to being 65 years or older; male gender (for HC); having a school/college level of education; being unmarried, divorced, or widowed; having a history of smoking; and avoiding alcohol use.

Conclusions

Heart disease and sociodemographic and lifestyle factors are substantially associated with a high score in all obesity indicators. The findings of this study are important because they can assist healthcare providers in implementing different therapies to prevent high BMI, WC, HC, and WHR.

## Introduction

Heart disease is one of the top causes of death worldwide [1,2]. Around 17.9 million people were responsible for 32% of all deaths worldwide in 2019 [3]. According to the most recent study, one in four Americans (approximately 659,000 people) aged over 40 will develop an irregular heartbeat, notably atrial fibrillation, which results in 15% of all strokes and dramatically raises the risk of death [4]. According to a previous study [5], age-adjusted all-cause mortality in Saudi Arabia declined from 740.8 in 2010 to 634.9 per 100,000 people in 2017, primarily due to cardiovascular illnesses. Nonetheless, cardiovascular illness was primarily responsible for the burden of disability.

Although most heart arrhythmias might not be harmful, they are frequent signs of severe cardiac problems that can be fatal [6]. As a result, diagnosing heart arrhythmias when they first appear is crucial. A previous study proposed an approach that can accurately identify a heart murmur and classify up to four cardiovascular diseases with a modest precision of 92.6% [7]. According to the American Heart Association (AHA), cardiovascular disease will be the top cause of mortality worldwide by 2030 and responsible for more than 23.6 million deaths [1]. This estimate implies that a heart attack will occur in America every 40 seconds. Additionally, according to data from the National Health and Nutrition Examination Survey, 6.2 million American adults ≥20 years of age experienced heart failure between 2013 and 2016 compared to 5.7 million between 2009 and 2012 [1].

Obesity, measured by the waist, hips, and body mass index (BMI) [8], is a significant risk factor for heart disease [4,9]. Approximately half of Americans (47%) have at least one of these obesity indicators, regardless of other sociodemographic, lifestyle, and clinical factors that increase the risk of heart disease [4]. A recent study indicated that heart disease was more likely to affect obese Australian individuals [10]. The economic toll of heart disease in Saudi Arabia is disproportionately high [11], although heart disease has increased. According to a recent study [12], Saudi Arabia’s overall health spending increased by $3.8 billion (4.3%) in 2019 due to rising rates of overweight and obesity-related non-communicable diseases, particularly cardiovascular diseases.

Several studies [1,13] have examined the relationship between obesity and health consequences. Although not fully understood, there may be a connection between heart disease and an increase in all forms of obesity. This study aimed to address this gap in the literature, with the central hypothesis that heart diseases, sociodemographic, and lifestyle factors might be associated with higher body mass index (BMI), waist circumference (WC), hip circumference (HC), and waist-to-hip ratio (WHR ) among adults.

## Materials and methods

Study design and setting

This cross-sectional study used data from the Midlife Development in the United States (MIDUS) refresher databases, which are accessible to the general public [14]. A nationally representative sample of 3,577 persons aged between 25 and 74 was recruited for the MIDUS refresher. This study collected demographic and biomarker data using an initial structured telephone interview and a clinic visit. The MIDUS refresher was a multidisciplinary project that examined psychosocial variables and health in individuals. All eligible respondents across sample types were subjected to a weighted response rate for the telephone interview and clinic visit to produce unbiased estimates by including them in the analysis. More resources detail the recruitment and evaluation processes for the MIDUS refresher [14].

Data access adheres to the National Archive of Computerized Data on Aging’s privacy and protection regulations. Harvard University, Georgetown University, the University of California at Los Angeles, the University of Wisconsin institutional review boards, and the University of California at Los Angeles authorized the MIDUS refresher. All MIDUS refresher participants provided written informed consent. Our study was exempted by King Saud University IRB Ethical Committee (E-23-7463).

Participants

Regardless of race or ethnicity, we included data from 3,574 people aged 25 to 74 who participated in the 2011-2014 National Survey of MIDUS refreshers. According to the presence or absence of heart disease, participants were divided into the following two groups: with and without heart disease. People with heart diseases, such as an irregular heartbeat (arrhythmia), a heart murmur, a heart attack or myocardial infarction (MI), or heart failure (HF), were included in the heart disease group. In contrast, the group without heart disease comprised individuals with no heart disease. Participants with missing data (n = 3) were excluded.

Exposure

Self-reported questions were used to evaluate patients with and without heart disease, such as arrhythmia, heart murmur, MI, and HF. The first question was, “Have you ever had heart trouble confirmed by a doctor?.” If the participants responded “yes,” they were asked the following question: “What was the diagnosis?.” If they replied “no” to the initial inquiry, it was assumed they did not have heart disease. Heart disease was considered if people mentioned any of the aforementioned cardiac disorders. Data were deemed missing when people responded “don’t know or not sure” to the first question or “refused” to answer it.

Outcomes

The outcome of interest was heart diseases and their relationship to adult obesity indicators, such as BMI, WC, HC, and WHR. In both men and women, BMI ≥30 kg/m^2^ [15], WC >102 cm (40 inches) and >88 cm (35 inches), and WHR >0.9 and >0.85, respectively, were all considered to be forms of obesity [16]. We estimated the participants’ BMI by dividing their weight in kilograms (kg) by the square of their height in meters (m). To get the height in m, the height measurement (in inches) was multiplied by 0.02. The weight in kg was converted by multiplying the weight (in pounds) by 0.45. Any heights higher than 84 inches were set to 84 inches to prevent extremes. The WC and HC were evaluated using the following questions in the 2011-2014 MIDUS refresher: “What is your waist size, that is, how many inches are around your waist?” The participants were instructed to measure their waists at the level of their navel at the vastest point. “What is your hip size, that is, how many inches do your hips measure at the widest point?” Measured at the widest point between your thighs and waist. People were instructed to use a non-stretchable tape to measure themselves while standing to find the answers to the abovementioned questions. Participants were told not to measure over thin clothing or other objects and to try to note their measurements to the nearest quarter inch (1/4 inch). The WC (in inches) was divided by the HC (in inches) to calculate the WHR.

Covariates

A series of sociodemographic and health behaviors were included, such as age, sex, race, education, marital status, employment, smoking, and alcohol intake. All these controlled variables were dichotomized as <65 years (reference) and ≥65 years; male and female (reference); white (reference) and minorities (black, mixed, Asian, and others); school/college and graduates (reference); married (reference) and unmarried/divorced/widowed; employed and unemployed (reference); smoker and non-smoker (reference); and alcohol consumption (yes and no). Consumption of alcohol was used as a reference. We used the threshold age of <65 and ≥65 years because the elderly and aging populations are particularly prone to cardiovascular diseases [17].

Statistical analysis

The Farrington-Manning Score test was used to determine the required sample size for each group to establish accurate results by applying the level of significance (alpha = 0.05), power (0.8), and proportion between groups (0.32, 0.21) [18]. The required minimum sample per group was 150. The Shapiro-Wilk test assessed the normality of the data [19]. Descriptive statistics were exhibited for constant and definite variables, including mean (standard deviation) and number (%). The typical distribution of anthropometric markers, such as BMI, WC, HC, and WHR, was shown for individuals with and without heart disease. The chi-square analysis and an independent Student’s t-test for categorical and continuous variables were used to determine the statistical significance between individuals without and with heart disease.

The individual relationships between heart disease, BMI, WC, HC, and WHR were predicted using unadjusted and adjusted linear regression models compared to individuals without heart disease. Model 1 was left unadjusted by including obesity indicators and heart disease status. Model 2 was adjusted for age, sex, race, education, marital status, employment status, smoking, and alcohol use, along with model 1. Estimates with corresponding standard errors (SEs) were computed for each model. All statistical analyses were conducted using Stata 14.1 statistical software (StataCorp, College Station, TX, USA). A p-value less than 0.05 was deemed statistically significant.

## Results

Participant characteristics

**Figure 1:**
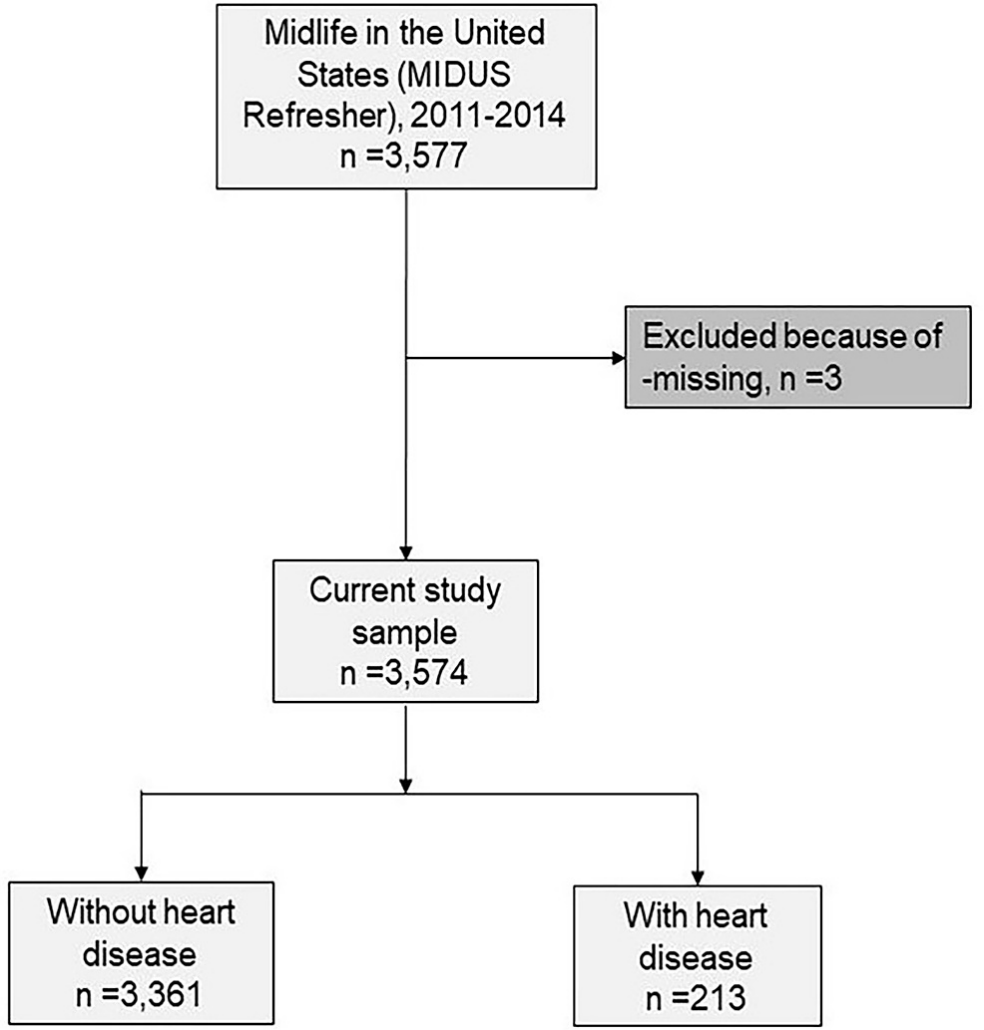
The flowchart of the study sample.

In this study, we removed missing data (n = 3) and used data from 3,574 of the 3,577 participants. There were 213 heart disease patients and 3,361 persons without heart disease (Figure 1). The patient characteristics are shown in Table 1. Among the 3,574 participants, 6% had heart disease, and 94% were heart disease-free. There were substantial differences in characteristics between individuals with cardiac disease and those without. However, whites and minorities did not significantly differ in the prevalence of heart disease.

**Table 1:** Characteristics of the study sample with and without heart disease. *: Black, mixed, Asian, and others.

Characteristics	Total, n = 3,574	Without heart disease, n = 3,361 (94%)	With heart disease, n = 213 (6%)	P-value
Age (year), mean (SD)	55.0 (13.3)	49.9 (14.2)	60.2 (12.5)	<0.001
Age group, n (%)				<0.001
<65 years	2,783 (77.9)	2,672 (96)	111 (4)	
≥65 years	791 (22.1)	689 (87)	102 (13)	
Sex, n (%)				0.009
Male	1,720 (48.1)	1,599 (93)	121 (7)	
Female	1,854 (51.9)	1,762 (95)	92 (5)	
Race, n (%)				0.403
White	2,924 (82.4)	2,744 (93.8)	180 (6.2)	
Minorities*	625 (17.6)	592 (94.7)	33 (5.3)	
Educational level, n (%)				0.003
School/college	1,645 (46.1)	1,788 (93)	135 (7)	
Graduates	1,923 (53.9)	1,568 (95.3)	77 (4.7)	
Marital status, n (%)				0.023
Married	2,282 (64)	2,161 (94.7)	121 (5.3)	
Unmarried/divorced/widow	1,282 (36)	1,190 (92.8)	92 (7.2)	
Employment status, n (%)				<0.001
Employed	2,278 (64)	2,176 (95.5)	102 (4.5)	
Unemployed	1,283 (36)	1,173 (91.4)	110 (8.6)	
Smoking status, n (%)				0.003
Smoker	1,520 (63.5)	1,398 (92)	122 (8)	
Non-smoker	873 (36.5)	836 (95.8)	37 (4.2)	
Alcohol intake, n (%)				0.040
Yes	1,920 (74)	1,809 (94.2)	111 (5.8)	
No	673 (25.9)	619 (92)	54 (8)	

**Figure 2:**
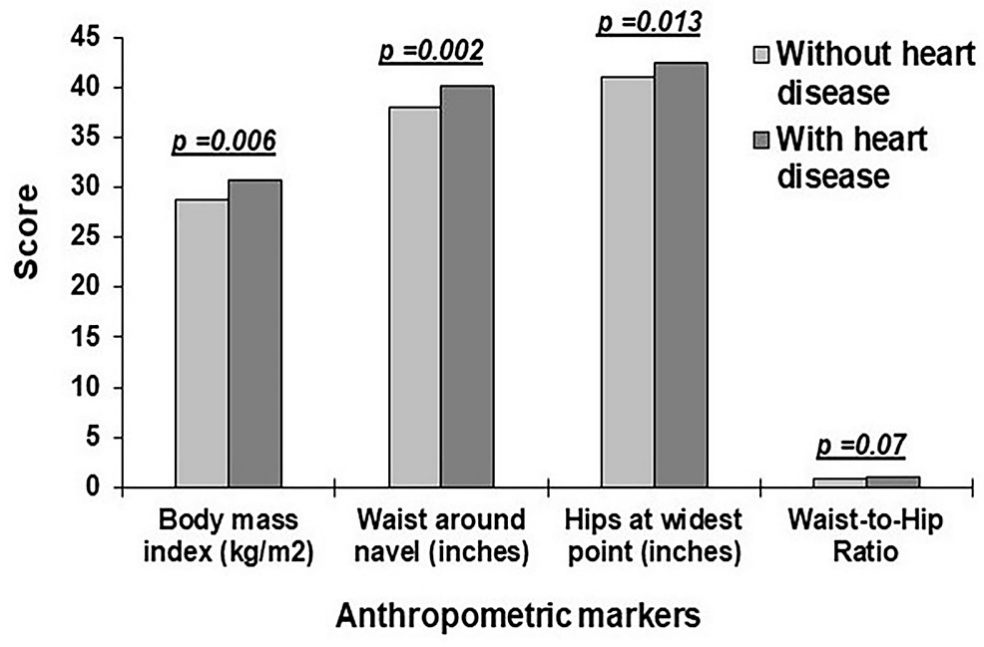
The distribution of average obesity indicators by cardiac disease and those without it.

Figure 2 presents the distribution of average BMI, WC, HC, and WHR scores for people with and without heart diseases. All anthropometric measurements differed considerably between individuals with and without heart disease groups. However, WHR did not differ substantially between the groups.

Association between heart diseases and body mass index

Individuals with heart diseases were significantly associated with a 1.32 kg/m^2^ increase in BMI compared to those without heart disease (29.42 kg/m^2^). Furthermore, individuals with heart disease were substantially associated with an increase in BMI of 1.12 kg/m^2^ after adjusting for covariates. Having a high school or college education and being unmarried, divorced, or widowed were each individually associated with an increased BMI of 1.47 kg/m^2^ and 0.52 kg/m^2^, respectively. Additionally, a 0.95 kg/m^2^ increase in BMI was strongly associated with individuals who did not drink alcohol (Table 2).

**Table 2:** The relationship between heart disease and body mass index. *p < 0.001; **p = 0.001; ***p < 0.05; ^#^: African Americans, Asians, or other non-whites.

Variable	Model 1		Model 2	
	β	SE	β	SE
Intercept	29.42	0.21	29.03	0.31
With versus without heart diseases	1.32**	0.38	1.12**	0.43
≥65 years versus <65 years			-0.002	0.26
Male versus female			-0.109	0.22
Minorities^#^ versus white			0.49	0.32
School/college versus graduate			1.47*	0.22
Unmarried/divorced/widow versus married			0.52***	0.23
Employed versus unemployed			0.04	0.24
Smoker versus non-smoker			0.26	0.23
No alcohol intake versus alcohol intake			0.95**	0.27

Association between heart diseases and waist circumference

Compared to those without heart disease (38.8 inches), people with heart disease exhibited substantial 1.14-inch growth in WC. After controlling for variables, heart disease remained substantially associated with a 0.63-inch gain in WC. WC increases of 0.62, 1.39, and 1.31 inches were linked independently to being male, 65 years or older, and attending school or college. Additionally, a 0.59- and 0.99-inch increase in WC was substantially correlated with smoking and avoiding alcohol, respectively (Table 3).

**Table 3:** Association between heart disease and waist circumference. *p < 0.001; **p = 0.001; ***p < 0.05; ^#^: African Americans, Asians, or other non-whites.

Variable	Model 1		Model 2	
	β	SE	β	SE
Intercept	38.8	0.20	37.9	0.30
With versus without heart diseases	1.14**	0.38	0.63***	0.42
≥65 years versus <65 years			0.62***	0.25
Male versus female			1.39*	0.22
Minorities^#^ versus white			0.04	0.32
School/college versus graduate			1.31*	0.22
Unmarried/divorced/widow versus married			0.41	0.23
Employed versus unemployed			-0.02	0.23
Smoker versus non-smoker			0.59***	0.23
No alcohol intake versus alcohol intake			0.99**	0.27

Association between heart diseases and hip circumference

A 0.94-inch increase in HC was more common in people with heart disease than those without (41.5 inches). After controlling for all variables, heart disease was substantially related to a 0.81-inch increase in HC. Age over 65, female gender, and not drinking alcohol were all associated with the rise in HC of 0.93, 1.54, and 0.86 inches, respectively (Table 4).

**Table 4:** Association between heart disease and hip circumference. *p < 0.001; **p = 0.001; ***p < 0.05; ^#^: African Americans, Asians, or other non-whites.

Variable	Model 1		Model 2	
	β	SE	β	SE
Intercept	41.5	0.21	41.8	0.31
With versus without heart diseases	0.94***	0.38	0.81***	0.41
≥65 years versus <65 years			0.93**	0.25
Female versus male			1.54*	0.22
Minorities^#^ versus white			-0.03	0.32
School/college versus graduate			0.38	0.22
Unmarried/divorced/widow versus married			0.01	0.23
Employed versus unemployed			0.14	0.23
Smoker versus non-smoker			0.15	0.23
No alcohol intake versus alcohol intake			0.86**	0.26

Association between heart diseases and waist-to-hip ratio

After controlling for all variables, individuals with heart disease had a 0.03 greater WHR than those without heart disease (WHR = 0.92), although this difference was not statistically significant (p = 0.821). A greater WHR was correlated with age 65 or above, being male, having completed high school or college, and being unmarried, divorced, or widowed, in that order (Table 5).

**Table 5:** Association between heart disease and waist-to-hip ratio. *p < 0.001; **p = 0.001; ***p < 0.05; #: African Americans, Asians, or other non-whites.

Variable	Model 1		Model 2	
	β	SE	β	SE
Intercept	0.94	0.005	0.92	0.007
With versus without heart diseases	0.01	0.009	0.03	0.009
≥65 years versus <65 years			0.01***	0.005
Male versus female			0.07*	0.005
Minorities^#^ versus white			0.07	0.007
School/college versus graduate			0.03*	0.005
Unmarried/divorced/widow versus married			0.01**	0.005
Employed versus unemployed			0.05	0.005
Smoker versus non-smoker			0.08	0.005
No alcohol intake versus alcohol intake			0.01	0.006

## Discussion

This study examined the relationship between heart diseases and BMI, WC, HC, and WHR indices of obesity as well as sociodemographic and lifestyle factors. According to the findings of this study, heart disease was significantly associated with an increase in BMI, WC, and HC. A rise in WC, HC, and WHR was strongly correlated independently with being 65 years of age or older. The male gender was associated significantly with higher WC and WHR while being female was strongly associated independently with rising HC. A school or college degree was independently related to a rise in BMI, WC, and WHR. Unmarried, divorced, or widowed status was strongly correlated independently with rising BMI and WHR. There was a significant increase in WC, HC, and BMI when alcohol use was absent, and smoking was only strongly associated with an increase in WC.

This study is the first to examine the relationship between heart disease [1,20] and a rise in obesity-related variables such as BMI [21], WC [22], and HC [23]. According to the AHA, it might result from failing to exercise regularly after a heart attack [24]. The AHA recommends that patients with a heart attack gradually increase their walking time to 30 minutes over several weeks, starting with 5-10 minutes daily. Additionally, our findings contradict those of previous research involving women aged 30 or older with no cardiovascular disease at baseline, in which increased WC was linked to a higher risk of coronary heart disease (CHD) [25]. Another study [26] indicated that a higher BMI reduces the likelihood of developing CHD in the future. Although the increase in WHR in this study was not statistically significant, it generally supports the findings of another study, in which WHR was more correlated with coronary vascular disease.

This result largely corroborates previous findings showing a relationship between sociodemographic and lifestyle parameters and BMI, WC, HC, and WHR [27]. According to a previous study [28], men and older adults have higher rates of WC than women and younger individuals. In earlier investigations, the lowest education level was linked to a higher BMI [29] and WC [29]. Furthermore, a previous study [30] revealed a more remarkable mean rise in BMI and WC in married women and men compared to unmarried individuals, contrasting with the present results. The results of this study provide additional evidence to support other studies showing a more significant rise (3.6 cm) in HC among females over five years [31]. A previous study demonstrated that smoking is independently linked to increased WC, as in this study [32]. Additionally, evidence from prior research indicates that decreases in WC and alcohol consumption among males are related [33].

Our preliminary findings suggest that arrhythmia, heart murmur, MI, and HF are associated with increased obesity indicators such as BMI, WC, HC, and WHR which can be altered. Obesity indicators easily collect measurements with a wide range of applications in pediatric, athletic, and adult populations, and there are no known limitations to their effectiveness [34]. Individuals can be measured to determine their body composition and assess their health, nutritional status, obesity, and risk for future illness [34]. One study evaluated anthropometric measurements such as BMI, WC, WHR, and waist-to-height ratio, determining that obesity is the most effective assessment. However, this conclusion is only sometimes accepted, and insufficient evidence supports one measurement technique.

Despite an obesity indicator error [35], our findings have significant implications for various therapies, weight loss, counseling, and exercises to reduce BMI, WC, HC, and WHR for preventing cardiovascular risk and mortality in adults. An earlier review detailed the global epidemic of obesity and the coherent action to tackle this issue [36]. Furthermore, the American College of Preventive Medicine has presented a practice policy report on weight management counseling of overweight adults based on a review of the current literature and recommendations [37]. Clinicians should always act jointly to obtain reproducible results that can be used in clinical settings to enhance long-term patient outcomes in adult populations. This will help them recognize at-risk individuals earlier and assist clinicians in encouraging a healthy lifestyle for at-risk patients to avoid the adverse effects of obesity.

Strengths and limitations

The study’s main strength is that it examined the relationship between obesity indicators as outcomes and heart disease indicators as exposure. The findings from this study are the first to reveal that heart diseases (such as arrhythmia, heart murmur, MI, and HF) are associated with increased modifiable obesity indicators such as BMI, WC, HC, and WHR. The study’s outcome measures, including BMI, WC, HC, and WHR, have been evaluated and shown to be reliable, as suggested by a previous study [16]. The higher sample size and the adoption of American standards for obesity indicators in this study were further strengths [38].

There were some limitations of this research. First, only a single time point was allowed for the cross-sectional design. Consequently, the results of this study are unable to establish causal links. Second, self-reporting was used to prove whether heart disease existed. Third, participants took their measurements of WC and HC. Due to the possibility of self-measurement errors, measurement bias may arise compared to those measured by a trained professional. It is challenging to predict how an illness will evolve, and this may also introduce bias and result in incorrect classification [39]. Fourth, the results cannot be extrapolated to the United States because they were based on a single MIDUS refresh database sample.

## Conclusions

Patients with heart disease patients have considerably higher BMI, WC, and HC than those without such conditions. Being 65 years or older; having low levels of education; being single, divorced, or widowed; and having a history of smoking were all independently associated with a high score in one or more obesity indicators. The study findings are significant because they will assist therapists in implementing various therapies, weight loss strategies, counseling, and exercise programs to reduce BMI, WC, HC, and WHR, and, subsequently, cardiovascular risk and mortality.
